# Molecular insights into *ANPEP* in gastric adenocarcinoma

**DOI:** 10.1590/1678-4685-GMB-2025-0110

**Published:** 2026-07-20

**Authors:** Taíssa Maíra Thomaz Araújo, Bianca de Fátima dos Reis Rodrigues, Jessica Manoelli Costa da Silva, Myrth Soares do Nascimento Remígio, Fabiano Cordeiro Moreira, Samir Mansour Moares Casseb, Williams Fernandes Barra, Geraldo Ishak, Ana Karyssa Mendes Anaissi, Leandro Magalhães, Amanda Vidal, Ronald Matheus da Silva Mourão, Eliel Barbosa Teixeira, Diego Pereira, Valéria Cristiane Santos da Silva, Daniel de Souza Avelar, Rubem Ferreira Silva, Ândrea Kely Ribeiro dos Santos, Samia Demachki, Livia Erika Carlos Marques, Rommel Rodriguez Burbano, Paulo Pimentel de Assumpção

**Affiliations:** 1 Universidade Federal do Pará (UFPA), Núcleo de Pesquisas em Oncologia (NPO), Belém, PA, Brazil.; 2 Hospital Ophir Loyola, Laboratório de Biologia Molecular, Belém, PA, Brazil.; 3 Universidade Federal do Pará (UFPA), Instituto de Ciências Biológicas, Laboratório de Genética Humana e Médica (LGHM), Belém, PA, Brazil.; 4 Instituto Tecnológico Vale, Belém, PA, Brazil.

**Keywords:** ANPEP, gastric adenocarcinoma, tumor microenvironment, biomarker

## Abstract

*Alanyl aminopeptidase* (*ANPEP*) has been implicated in various cancers, but its specific role in gastric adenocarcinoma (GC) remains incompletely understood. This study analyzed *ANPEP* gene expression in gastric cancer (GC), peritumoral tissue (PTT), metaplasia (M), and normal tissue (N). Total RNA was extracted, libraries were prepared and sequenced on the Illumina NextSeq 500. Data were processed using the nf-core/rnaseq pipeline. Transcript quantifications were imported with tximport and normalized using DESeq2. Differential expression (|log₂FC| > 1; adj. p < 0.05) and Kruskal-Wallis tests identified key genes. *ANPEP* was significantly upregulated in GC, PTT, and M compared to normal tissue (p < 0.01), suggesting its involvement in early mucosal transformation and malignant progression. Heatmap and pathway enrichment analysis revealed upregulation of genes related to immune function and oxidative stress, indicating an immunosuppressive and apoptosis-resistant tumor microenvironment. Correlation analyses identified strong positive associations between *ANPEP* and genes involved in cytoskeletal remodeling, immune modulation, and metabolic regulation, suggesting that *ANPEP* supports both the invasive potential of tumor cells and the establishment of an immunosuppressive, therapy-resistant niche. These findings position *ANPEP* as a promising biomarker for early detection and a candidate for targeted therapies.

## Introduction

Gastric cancer (GC) remains a significant global health challenge, ranking among the leading causes of cancer-related mortality worldwide (Sung et al., 2021). Despite advances in early detection and therapeutic strategies, the molecular mechanisms underlying GC progression and metastasis are still incompletely understood, necessitating the identification of novel biomarkers and therapeutic targets (Matsuoka and Yashiro, 2024). Among the genes implicated in GC pathogenesis, *ANPEP*/CD13 (Aminopeptidase N, also known as CD13) has emerged as a promising candidate due to its multifaceted roles in tumor biology (Wickström et al., 2011).

*ANPEP* encodes a zinc-dependent metalloprotease expressed on the surface of various cell types, including epithelial and endothelial cells, where it regulates processes such as cell migration, invasion, and angiogenesis (Lendeckel et al., 2023). In the context of cancer, *ANPEP* is frequently overexpressed in tumor tissues and is associated with aggressive phenotypes, including enhanced metastatic potential and resistance to therapy (Hu et al., 2020; Xiu et al., 2022). Recent studies have highlighted its significance in gastrointestinal malignancies, particularly GC, where *ANPEP* overexpression correlates with poor prognosis and advanced disease stages (Zhang et al., 2023).

For instance, Xiu et al. (2022) demonstrated that *ANPEP* promotes tumor cell invasion in GC by modulating the extracellular matrix and facilitating epithelial-to-mesenchymal transition (EMT), a critical step in metastasis. 

The interplay between *ANPEP* and signaling pathways, such as Wnt/β-catenin and PI3K/Akt, further underscores its relevance as a therapeutic target (Wang et al., 2015; Guo et al., 2019). Studies have identified CD13 as a CSC-specific membrane marker, including for hepatocellular carcinoma (HCC), and cholangiocarcinoma (Laukkanen and Castellone, 2016; Cui et al., 2018). From a functional point of view, CD13 is known to protect CSC from apoptosis (Kesavardhana and Kanneganti, 2018). Pharmacological inhibition of CD13 enzymatic activity can enhance the antitumor effects of chemotherapeutic agents, e.g., 5-fluorouracil (Sun et al., 2015; Xiu et al., 2022).

Hoft et al. (2024) reported that identification of a metaplastic cell expressing the cancer-associated biomarker *ANPEP*, present in *Helicobacter pylori*-induced gastritis and autoimmune atrophic gastritis, indicates the carcinogenic capacity of both diseases. This finding may guide early detection and risk stratification for patients with chronic gastritis.

This study aims to consolidate current knowledge on the role of *ANPEP* in gastric cancer, focusing on its molecular mechanisms, clinical implications, and potential as a therapeutic target. By synthesizing findings from recent studies, we seek to provide insights into how *ANPEP* contributes to GC progression and highlight avenues for future research and clinical translation.

## Subjects and Methods

### Ethical approval and patient selection

This study was approved by the Research Ethics Committee (CEP) under protocol number CAAE 47580121.9.0000.5634. All enrolled participants provided written informed consent in accordance with the Declaration of Helsinki. Samples of tumor tissue (156) and peritumoral tissue (PTT) (186) were collected from patients with histologically confirmed gastric adenocarcinoma (GAC). In addition to these, samples from intestinal metaplasia (M) (20) and normal tissue samples (N) (27) were also included for comparative purposes.

### RNA extraction and sequencing

Total RNA was extracted using the TRIzol^®^ reagent (Invitrogen), following the manufacturer’s protocol. RNA quality and integrity were verified prior to library preparation. RNA concentration was determined using the Qubit 4.0 fluorometer, purity was evaluated with the NanoDrop ND-1000 spectrophotometer (Thermo Fisher Scientific), and integrity was analyzed using the TapeStation 2200 system (Agilent). Libraries were constructed using the TruSeq Stranded Total RNA kit (Illumina^®^), and sequencing was performed in paired-end mode on the Illumina NextSeq 500^®^ platform.

### Dry Lab Pipeline nf-core/rnaseq

RNA sequencing data were processed using the nf-core/rnaseq pipeline (Ewels et al., 2020), a community-curated workflow that ensures reproducibility and consistency in RNA-seq analyses. The pipeline performs quality control (QC), adapter trimming, and read alignment or pseudo-alignment using STAR and Salmon, respectively. The final outputs include a gene-level expression matrix.

### Import and normalization of quantified reads

Transcript-level quantifications generated by Salmon were imported into the R statistical environment (R Core Team, 2018) using the tximport package (Soneson et al., 2015). This step enables aggregation of transcript abundances to the gene level while correcting for transcript length and library size. The resulting count matrices were normalized using the DESeq2 package, which estimates size factors to account for differences in sequencing depth and sample composition. Normalized counts were extracted for downstream analyses.

### Differential expression analysis

Differential gene expression analysis was performed between tissue conditions using DESeq2. Genes were considered differentially expressed (DE) if they exhibited an absolute log₂ fold-change greater than 1 and an adjusted p-value (Benjamini-Hochberg correction) below 0.05. *ANPEP* expression levels across multiple groups were evaluated using the Kruskal-Wallis test, followed by Dunn’s post-hoc test for pairwise comparisons between clinical characteristics. In all instances, statistical significance was defined as p < 0.05.

### Co-expression analysis and gene prioritization

Co-expression analysis was restricted to tumor samples (GAC, n=156) to identify genes whose expression pattern mirrors that of *ANPEP* in the tumor microenvironment. Spearman correlation coefficients (ρ) between *ANPEP* and all other expressed genes were computed, with p-values adjusted by the Benjamini-Hochberg method. Only genes with padj < 0.05 were retained. Biologically relevant candidates were selected using a stringent threshold of ∣ρ∣ ≥ μ + 2σ, where μ and σ denote the mean and standard deviation of the ρ distribution between ANPEP and all other expressed genes.

### Co-expression network visualization

To visualize the co-expression structure, a network was constructed using the top 30 genes with the highest absolute Spearman ρ with *ANPEP* (|ρ| ≥ 0.67). *ANPEP* was positioned as the central hub node, with edges connecting each gene to *ANPEP*. Edge width was proportional to |ρ|. Nodes were colored according to correlation direction (positive: red; negative: blue). The network was built and visualized using the igraph, tidygraph, and ggraph R packages.

### Single-sample gene set enrichment analysis (ssGSEA)

Functional pathway activity was estimated using single-sample Gene Set Enrichment Analysis (ssGSEA) implemented in the GSVA package (version 2.0.7; Hänzelmann et al., 2013). The input expression matrix consisted of log2 (normalized counts + 1) values for all samples (N=389). Gene sets were retrieved from the Molecular Signatures Database (MSigDB) C2 Reactome collection (*Homo sapiens*) using the msigdbr R package (2.4.1.0). To ensure biological relevance, we retained pathways above the 25^th^ variance percentile and removed redundancies (Pearson r > 0.95). Hierarchical clustering was performed using Ward.D2 linkage based on Kendall correlation distance, implemented via the pheatmap R package (1.0.12).

### Ethics statement

The studies involving humans were approved by Ethics Committee for Research of the João de Barros Barreto University Hospital (CAAE - 47580121.9.0000.5634). The studies were conducted in accordance with the local legislation and institutional requirements. The participants provided their written informed consent to participate in this study. Written informed consent was obtained from the individual(s) for the publication of any potentially identifiable images or data included in this article.

## Results

### Expression of *ANPEP*

**Figure 1 -:**
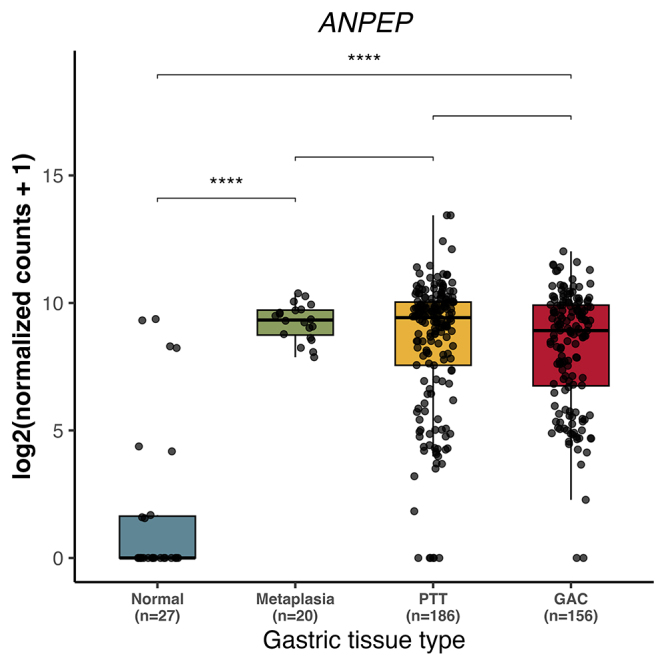
ANPEP expression across gastric tissue types. Boxplot showing log2-normalized expression of *ANPEP* in Normal (n=27), Metaplasia (n=20), Peritumoral (n=186), and Gastric Adenocarcinoma (GAC, n=156) tissues. Each dot represents an individual sample. Only significant comparisons are shown (**** p < 0.0001).

**Figure 2 -:**
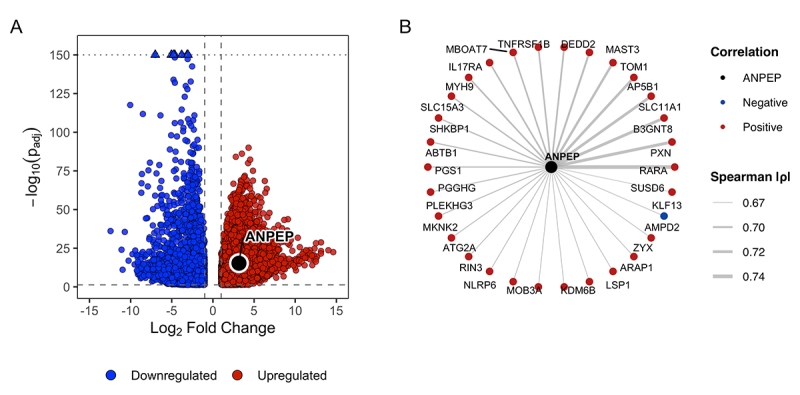
Differentially expressed genes and ANPEP co-expression network in gastric adenocarcinoma. (A) Volcano plot displaying 12,391 differentially expressed genes (DEGs) identified by DESeq2 (|Log₂ Fold Change| > 1 and adjusted p-value < 0.05, Benjamini-Hochberg) between tumor and normal gastric tissues. Upregulated genes are shown in red and downregulated in blue. ANPEP is highlighted in black. Eight genes with -log₁₀(p~adj~) > 150 are represented as triangles at the plot boundary. (B) Co-expression network showing the top 30 genes with highest Spearman correlation with ANPEP (|ρ| ≥ 0.67), identified exclusively in tumor samples. Edge width reflects correlation strength. Red nodes indicate positive correlation; blue nodes indicate negative correlation (KLF13).

Analysis of *ANPEP* gene expression was performed across a cohort of 389 samples, including gastric adenocarcinoma (GAC), peritumoral tissue (PTT), metaplasia (M) and normal tissue samples. *ANPEP* exhibited variable expression across sample types, with significantly higher expression in GAC, PTT, and M compared to N tissues. When evaluating *ANPEP* expression in paired tissue comparisons, upregulation in both metaplastic and malignant tissues relative to normal samples was observed (padj < 0.001; Dunn’s test), suggesting that *ANPEP* may be upregulated early during gastric mucosal transformation and remain elevated through to malignant progression (Figure 1 and 2A).

*ANPEP* expression levels showed significant associations with neoadjuvant therapy, metastatic status, and overall clinical staging. Regarding cTNM, post-hoc Dunn’s test identified that the significance was primarily driven by higher expression in Stage IV compared to Stage I (padj = 0.029) and Stage III (padj = 0.043). Conversely, no significant differences were observed for Lauren classification, EBV or *H. pylori* infection status, and tumor topography (Table 1).

**Table 1 -:** Association between ANPEP expression and clinicopathological variables in GAC.

Clinicopathological variable	p-value
Lauren classification	0.656
Neoadjuvant treatment	< 0.001
EBV infection	0.291
*H. pylori* infection	0.477
pT stage	0.342
pN stage	0.111
pM stage	0.007
Overall stage	0.028
Sex	0.562
Tumor topography	0.430

### Co-expression analysis

The correlation analysis involving the gene *ANPEP* revealed a set of genes whose expression patterns were positively associated with *ANPEP* across multiple tumor samples (Figure 2), many of which are implicated in key oncogenic processes. A total of 114 co-expressed genes were identified (Table S1), and the top 30 genes with the highest correlation (|ρ| ≥ 0.67) were selected. Among the genes analyzed, all exhibited positive correlations with *ANPEP*, except for *KLF13,* which was negatively associated. Additionally, *RARA, PXN, B3GNT8, SLC11A1,* and *AP5B1* stood out due to the strength of their correlations (|ρ| ≥ 0.74) (Figure 2 B ).

Among the co-expressed genes, *TNFRSF1B*, *VSIR*, and *SIRPA* are immune-related genes involved in immune evasion, T cell suppression, and regulation of inflammatory responses. Additionally, *NCF4* (a component of the *NADPH* oxidase complex) is involved in reactive oxygen species (ROS) production, linking oxidative stress to tumor progression and resistance to cell death. Several solute carrier genes, such as *SLC25A37*, *SLC16A3*, and *SLC11A1*, highlight metabolic reprogramming, particularly in mitochondrial iron transport and lactate export, essential for sustaining cancer cell proliferation. Transcriptional regulators like *SPI1* and *RARA*, underscore the role of epigenetic and transcriptional dysregulation in GC pathogenesis. Additionally, the gene *DEDD2*, associated with cell death pathways, may indicate adaptations that modulate apoptosis. 

**Figure 3 -:**
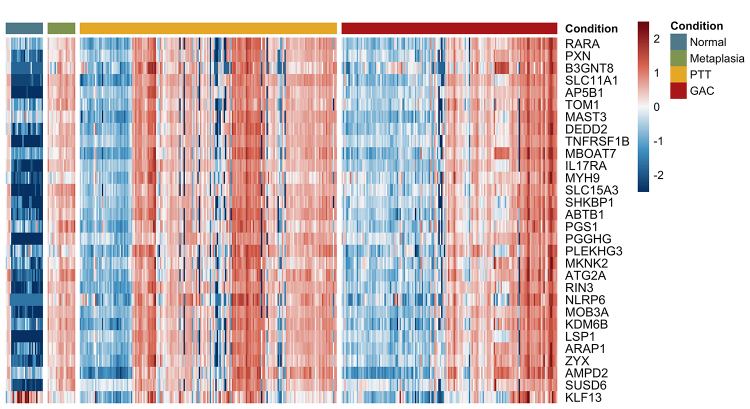
Expression profiles of the top 30 ANPEP co-expressed genes across gastric tissue types. Heatmap displaying z-score normalized log2 expression of the 30 genes with highest Spearman correlation with ANPEP (|ρ| ≥ 0.67), identified from 114 co-expressed genes in tumor samples. Rows are ordered by decreasing Spearman ρ (RARA, ρ = 0.74, at top; KLF13, ρ = −0.67, at bottom). Columns represent individual samples clustered within each condition using Ward.D2 linkage based on Kendall correlation distance. Conditions are shown left to right: Normal (n=27), Metaplasia (n=20), Peritumoral (n=186), and GAC (n=156). Color scale represents z-scored expression (blue = low; red = high).

The heatmap revealed that genes positively co-expressed with *ANPEP* exhibited higher expression levels in GAC, PTT, and M tissues, while showing low expression in N samples. Despite this overall pattern, variability in gene expression across GAC, PTT, and M samples suggests the presence of tumor heterogeneity. In contrast, *KLF13,* a transcription factor, displayed an inverse pattern, with higher expression in N tissues and reduced expression in the remaining tissue types (Figure 3).

### Functional pathway activity

Pathway enrichment analysis based on the expression of the 114 genes co-expressed with *ANPEP* revealed that pathways related to the immune system, transcriptional activity, and oncogenic signaling were enriched in the predominant profile of GAC, PTT, and M samples. Despite this, pathways associated with tumor invasiveness and mesenchymal phenotype were found to have variable enrichment across samples, supporting the presence of intra-group heterogeneity.

**Figure 4 -:**
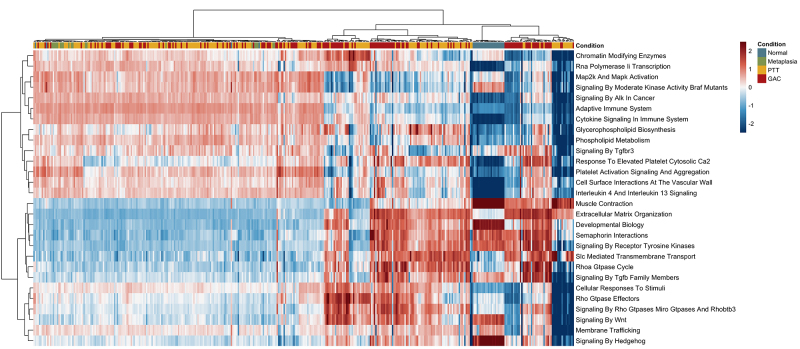
Functional pathway activity across gastric tissue types. Heatmap displaying ssGSEA enrichment scores (GSVA, R package) for 28 Reactome pathways derived from the 114 genes co-expressed with ANPEP in tumor samples. Scores were z-score normalized per pathway. Pathways were selected from the MSigDB C2 Reactome collection (Homo sapiens) requiring a minimum overlap of 3 genes, followed by removal of redundant pathways (Pearson r > 0.95) and retention of the top 75% by variance. Columns represent individual samples (Normal, n=27; Metaplasia, n=20; Peritumoral, n=186; GAC, n=156) and rows represent pathways. Both rows and columns were clustered using hierarchical clustering with Ward.D2 linkage based on Kendall correlation distance. Color scale represents z-scored ssGSEA enrichment (blue = low; red = high).

Enrichment scores were estimated for each sample across different tissue types based on the expression of the genes co-expressed with *ANPEP*. In Figure 4, clustering analysis revealed distinct subsets of samples. A substantial number of GAC, PTT, and M samples clustered together, showing similar pathway enrichment patterns. The lack of clear separation among these tissue types reinforces the presence of biological heterogeneity. Conversely, N samples displayed a more homogeneous profile, with overall lower levels of pathway enrichment compared to the other tissue types.

## Discussion

The present study provides compelling evidence for the multifaceted role of *ANPEP* in GC progression, highlighting its potential as both a biomarker and therapeutic target. The observed upregulation of *ANPEP* across GAC, PTT and M tissue compared to N tissues aligns with previous findings that link *ANPEP* overexpression to early mucosal transformation and malignant progression in GC (Hoft et al., 2024). 

Notably, *ANPEP* expression was associated with neoadjuvant therapy, metastasis, and clinical staging, with higher levels in advanced tumors, suggesting a possible role in tumor progression. The association with therapy may reflect the effects of treatment or characteristics of tumors undergoing this approach. Conversely, the absence of association with Lauren classification, EBV, *H. pylori*, and tumor location may indicate that *ANPEP* expression is more related to disease progression than to etiological or histopathological factors of the disease.

The correlation analysis revealed the upregulation of a subset of genes, many of which are implicated in key oncogenic processes. Notably, several solute carrier genes, such as *SLC25A37*, *SLC16A3*, and *SLC11A1*, highlight metabolic reprogramming, particularly in mitochondrial iron transport and lactate export, essential for sustaining cancer cell proliferation. Additionally, the positive correlation with *DEDD2*, associated with cell death pathways, may indicate adaptations that modulate apoptosis.

Additionally, the upregulation of a subset of genes was observed, comprising immune-related genes (e.g., *ITGB2*, *VSIR*, *IL17RA*, *TNFRSF1B, STAT6, TGFB1, NUMB*) and inflammation/oxidative stress genes (e.g., *NLRP6*, *NCF4*, *NLRP12, NFKB2, G6PD, FTH1, ATG16L2*) - further elucidates *ANPEP*’s contribution to an immunosuppressive tumor microenvironment (TME) (Zhang et al., 2017). In particular, *SIRPA*, an inhibitory immune receptor, was also positively correlated with *ANPEP*. This gene is known to inhibit phagocytosis by macrophages, potentially aiding tumor cells in escaping immune surveillance (Mhaidly and Mechta-Grigoriou, 2020). This association implies a functional connection between *ANPEP* expression and the regulation of innate immune responses, including macrophage activation and immune evasion.

In this context, *VSIR* (also known as *VISTA*) is a negative immune checkpoint regulator (Le Mercier et al., 2014) that showed strong co-expression with *ANPEP*, suggesting a possible synergistic role in suppressing adaptive immune responses and promoting immune tolerance within the tumor, consistent with its role in creating an immunosuppressive microenvironment (Huang et al., 2024). This observation supports the notion of a tumor phenotype with reduced immune surveillance (Huang *et al*., 2024) and is further corroborated by studies showing that upregulation of immune checkpoint pathways enhances tumor immune evasion, a hallmark of advanced GC (Neo and Lundqvist, 2020).

The link between *ANPEP* and oxidative stress reinforces its role in tumor progression. The *NCF4* gene, positively correlated with *ANPEP* expression, is a component of the NADPH complex that is essential for the generation of ROS. Increased ROS levels can promote DNA damage, genomic instability, and activate signaling pathways that enhance tumor cell proliferation and survival. Specifically, *NOX4*, a member of the NADPH oxidase family, has been shown to regulate GC cell proliferation and apoptosis through the *GLI1* pathway, highlighting the role of ROS in GC pathogenesis (Tang et al., 2018).

In addition to immune and metabolic pathways, *ANPEP* co-expression was associated with genes involved in cytoskeletal organization and tumor invasiveness, such as *ZYX* (*Zyxin*), involved in actin cytoskeleton organization; *ARAP1*, which regulates cytoskeletal remodeling via ARF and Rho signaling pathways; and *CNN2* (Calponin 2), a protein associated with cell contraction and motility. Their co-expression with *ANPEP* reinforces its putative involvement in cytoskeletal regulation and tissue remodeling processes that are characteristic of invasive tumors, as cytoskeletal dynamics are crucial for tumor cell motility and metastasis (Guo et al., 2023). 

The co-expression of *ANPEP* with transcription factors and epigenetic regulators, including *SPI1*, *RARA*, *KLF13*, *HELLS*, and *KDM6B*, highlights the contribution of transcriptional and epigenetic dysregulation to GC pathogenesis. Among these regulators, *SPI1* has been implicated in promoting GC progression by activating the IL6/JAK2/STAT3 signaling pathway (Hou et al., 2023), supporting the functional relevance of this network. In contrast, the inverse expression pattern of *KLF13* suggests a potential regulatory imbalance, possibly reflecting loss of tumor-suppressive functions within this network. Together, these findings indicate that *ANPEP* is part of a broader regulatory axis integrating transcriptional control, immune modulation, and cellular adaptation.

Additional genes positively correlated with *ANPEP*, such as *MBP*, *MYH9*, *ATG2A*, *MCL1*, *TOM1*, *MAST3* and *B3GNT8* converge on central mechanisms of tumorigenesis, including cell survival, invasion and adaptation to the tumor microenvironment. These genes are predominantly upregulated in GAC, with the exception of *MBP*, which is downregulated. 

From a functional perspective, *MBP* has been described as a potential tumor suppressor, associated with the inhibition of growth and metastasis (Hsu et al., 2009; Lung et al., 2010), whereas *MYH9* acts as an oncoprotein, promoting tumor progression, invasion, metastasis and resistance to anoikis via pathways such as Wnt/β-catenin and EMT (Li et al., 2024; Zeng et al., 2025). Similarly, *ATG2A* regulates autophagy, contributing to the survival of tumor cells (Wu et al., 2024), while *MCL1*, an anti-apoptotic gene, promotes resistance to cell death and chemotherapy (Akagi et al., 2013). Furthermore, *TOM1* is involved in endosomal trafficking and ubiquitination, suggesting a role in tumor vesicular dynamics (NCBI, 2026a), and *MAST3*, a microtubule-associated serine/threonine kinase, is involved in cell signaling, with emerging evidence of its involvement in tumor processes (Deng et al., 2025). In parallel, *B3GNT8*, a glycosyltransferase, has been implicated in the progression of various types of cancer. In GC, its expression is associated with increased cell migration and invasion, possibly through the modulation of poly-lactosamine chains on surface glycoproteins, such as CD147 (Shen et al., 2017).

The *AP5B1* gene also showed a positive correlation with *ANPEP*. This gene encodes a subunit of the AP-5 adaptor complex, which is involved in intracellular transport, particularly in the sorting of endosomal cargo and in lysosomal function (NCBI, 2026a). Despite its importance in cellular homeostasis, there is no consistent evidence in the literature describing the role of *AP5B1* in cancer. Thus, the increase in its expression and its correlation with *ANPEP* observed in this study highlight a finding that remains largely unexplored in the oncological context.

Collectively, these findings support a model in which elevated *ANPEP* expression is part of a coordinated transcriptional program involving genes that promote immune modulation, cell migration, and metabolic adaptation, which represents hallmarks of tumor progression and immune evasion. This reinforces the notion that *ANPEP* contributes to a tumor-promoting TME and acts as a central player in GC biology, bridging molecular and immunological factors.

The upregulation of *ANPEP* in pre-malignant and malignant states, coupled with its association with an immunosuppressive and plastic TME, provides a molecular basis for its potential as a prognostic biomarker and therapeutic target. Targeted therapies, such as *ANPEP* inhibitors could be explored, particularly in combination with immune checkpoint inhibitors, to overcome the immunosuppressive barriers identified.

## Conclusion

This study demonstrates that *ANPEP* is consistently upregulated from early pre-malignant stages through to advanced gastric cancer. Its expression is tightly linked with a network of genes involved in immune suppression, oxidative stress, metabolic reprogramming, cytoskeletal remodeling, and cell survival pathways. These coordinated transcriptional changes suggest that *ANPEP* plays a central role in shaping an immunosuppressive, metabolically adaptive, and invasion-prone tumor microenvironment that facilitates gastric cancer progression and immune evasion. The strong associations between *ANPEP* and key immune checkpoint regulators (e.g., *VSIR and SIRPA*), inflammatory mediators (e.g., *TNFRSF1B and TGFB1*), metabolic and oxidative stress-related genes (e.g., *SLC16A3*, *SLC25A37*, and *NCF4*), as well as cytoskeletal components (e.g., *PXN*, *CNN2 and ZYX*) further highlight its multifaceted contribution to tumor biology. In addition, correlations with genes involved in cell survival and adaptation, such as *MCL1*, *ATG2A*, *MYH9*, and *B3GNT8*, reinforce its role in promoting tumor progression and plasticity. These findings position *ANPEP* as a promising biomarker for early detection and a potential therapeutic target, particularly in strategies aiming to disrupt tumor immune escape and enhance treatment efficacy in gastric cancer.

## Supplementary material

The following online material is available for this article:

Table S1 - Genes co-expressed with ANPEP in GAC.

## Data Availability

All relevant data is contained within the article: The original contributions presented in the study are included in the article, further inquiries can be directed to the corresponding author.
