# Supplementary material for: Molecular insights into ANPEP in gastric adenocarcinoma
Source: Genet Mol Biol. 2026 Jul 20;49(Suppl 4):e20250110. doi: 10.1590/1678-4685-GMB-2025-0110 (PMC13397915; doi:10.1590/1678-4685-GMB-2025-0110)
Supplement: Table S1 - [file 1415-4757-GMB-49-s4-e20250110-s1.pdf]

Genetics and Molecular Biology
https://doi.org/10.1590/1678-4685-GMB-2025-0110

Supplementary Material to "Molecular Insights into ANPEP in Gastric Adenocarcinoma"

Table S1 - Genes co-expressed with ANPEP in GAC.

Gene	ρ Spearman	log2FC	FC Management	Correlation with ANPEP	Functional Annotation
**ALPL**	0.635	3.602	**UP**	Positive	
**VSIR**	0.664	3.473	**UP**	Positive	Negative immune checkpoint (VISTA)
**NCF4**	0.635	3.370	**UP**	Positive	NADPH oxidase complex / ROS
**ITGB2**	0.667	3.311	**UP**	Positive	Leukocyte adhesion
**SLC16A3**	0.640	3.223	**UP**	Positive	Lactate exporter / Warburg
**ZYX**	0.671	3.213	**UP**	Positive	Organisation of the actin cytoskeleton
**TNFRSF1B**	0.695	3.018	**UP**	Positive	TNF signalling / inflammation
**IL17RA**	0.695	2.975	**UP**	Positive	IL-17 receptor / immune response
**LSP1**	0.672	2.963	**UP**	Positive	Leukocyte motility
**CNN2**	0.645	2.925	**UP**	Positive	Cell contraction and motility
**SLC11A1**	0.719	2.897	**UP**	Positive	Mitochondrial iron transport
**SPI1**	0.668	2.896	**UP**	Positive	Transcription factor / IL-6-JAK-2-

Gene	ρ Spearman	log2FC	FC Management	Correlation with ANPEP	Functional Annotation
					STAT3
**SIRPA**	0.662	2.772	**UP**	Positive	Inhibitory receptor / immune evasion
**PXN**	0.724	2.437	**UP**	Positive	Focal adhesion / tumour invasion
**ARAP1**	0.672	2.405	**UP**	Positive	ARF/Rho cytoskeletal remodelling
**DEDD2**	0.697	2.376	**UP**	Positive	Cell death pathway / apoptosis
**SLC25A37**	0.644	1.933	**UP**	Positive	Mitochondrial iron transport
**RARA**	0.741	1.833	**UP**	Positive	Retinoic acid receptor / transcriptional regulation
**MBP**	0.637	-7.008	**DOWN**	Positive	Highest FC magnitude in the set — downregulated
**NLRP6**	0.674	3.369	**UP**	Positive	Inflammasome / pyroptosis
**NLRP12**	0.633	3.115	**UP**	Positive	Downregulation of NF-κB
**STAT6**	0.641	2.888	**UP**	Positive	IL-4/IL-13 signalling / M2 polarisation
**ATG16L2**	0.664	2.648	**UP**	Positive	Autophagy
**TGFB1**	0.655	2.542	**UP**	Positive	Immunosuppression / TME / EMT
**MYH9**	0.694	2.087	**UP**	Positive	Myosin / tumour migration and invasion

Gene	ρ Spearman	log2FC	FC Management	Correlation with ANPEP	Functional Annotation
**HELLS**	-0.639	2.060	**UP**	Negative	Chromatin remodelling / epigenetics
**KDM6B**	0.673	1.981	**UP**	Positive	H3K27 demethylase / epigenetic regulation
**ATG2A**	0.675	1.879	**UP**	Positive	Autophagy
**MCL1**	0.651	1.824	**UP**	Positive	Anti-apoptotic / treatment resistance
**NFKB2**	0.632	1.784	**UP**	Positive	Nf-κB pathway / tumour survival
**G6PD**	0.635	1.771	**UP**	Positive	Pentose pathway / oxidative stress
**TOM1**	0.712	1.637	**UP**	Positive	Endosomal trafficking / ubiquitination
**FTH1**	0.658	1.480	**UP**	Positive	Ferritin / iron metabolism / ferroptosis
**NUMB**	0.652	1.438	**UP**	Positive	Tumour suppressor / Notch pathway
**MAST3**	0.703	1.236	**UP**	Positive	Kinase / cell signaling
**PSD4**	0.635	3.872	**UP**	Positive	
**SH3BGRL3**	0.633	3.858	**UP**	Positive	
**CCDC113**	-0.639	3.746	**UP**	Negative	
**PCDH7**	-0.649	-3.711	**DOWN**	Negative	

Gene	ρ Spearman	log2FC	FC Management	Correlation with ANPEP	Functional Annotation
**B3GNT8**	0.722	3.536	**UP**	Positive	
**PEAK3**	0.634	3.495	**UP**	Positive	
**MYADM**	0.638	3.472	**UP**	Positive	
**MOB3A**	0.673	3.393	**UP**	Positive	
**C5AR2**	0.644	3.336	**UP**	Positive	
**FRAT2**	0.636	3.293	**UP**	Positive	
**XKR8**	0.633	3.283	**UP**	Positive	
**GMIP**	0.651	3.249	**UP**	Positive	
**ABTB1**	0.685	3.244	**UP**	Positive	
**PGGHG**	0.679	3.236	**UP**	Positive	
**COBLL1**	-0.645	3.210	**UP**	Negative	
**IFITM2**	0.631	3.197	**UP**	Positive	
**FOLR3**	0.655	3.188	**UP**	Positive	
**LRRC25**	0.644	3.180	**UP**	Positive	
**ARHGAP27**	0.668	3.159	**UP**	Positive	
**TYK2**	0.632	3.111	**UP**	Positive	
**ARHGAP4**	0.652	3.055	**UP**	Positive	

Gene	ρ Spearman	log2FC	FC Management	Correlation with ANPEP	Functional Annotation
**UNC13D**	0.643	3.046	**UP**	Positive	
**NADK**	0.652	2.993	**UP**	Positive	
**PRAM1**	0.639	2.950	**UP**	Positive	
**AP5B1**	0.716	2.844	**UP**	Positive	
**RAB3D**	0.650	2.840	**UP**	Positive	
**PPCDC**	0.631	2.839	**UP**	Positive	
**LITAF**	0.641	2.817	**UP**	Positive	
**ZNF467**	0.647	2.771	**UP**	Positive	
**MBOAT7**	0.695	2.770	**UP**	Positive	
**DHX34**	0.647	2.761	**UP**	Positive	
**RIN3**	0.674	2.721	**UP**	Positive	
**LARP1B**	-0.630	2.715	**UP**	Negative	
**ZNF516**	0.658	2.714	**UP**	Positive	
**NBEAL2**	0.668	2.676	**UP**	Positive	
**CSRNP1**	0.636	2.664	**UP**	Positive	
**SH3BP2**	0.666	2.629	**UP**	Positive	
**TNC**	-0.634	2.625	**UP**	Negative	

Gene	ρ Spearman	log2FC	FC Management	Correlation with ANPEP	Functional Annotation
**FRAT1**	0.667	2.622	**UP**	Positive	
**CYP4F3**	0.651	2.596	**UP**	Positive	
**ELL**	0.631	2.575	**UP**	Positive	
**METTL15**	-0.632	2.525	**UP**	Negative	
**SHKBP1**	0.685	2.506	**UP**	Positive	
**ATXN7L3**	0.639	-2.448	**DOWN**	Positive	
**CAMK2G**	0.631	-2.407	**DOWN**	Positive	
**NUAK2**	0.637	2.393	**UP**	Positive	
**BCCIP**	-0.652	2.391	**UP**	Negative	
**SUSD6**	0.669	2.359	**UP**	Positive	
**PLCB2**	0.647	2.339	**UP**	Positive	
**TLN1**	0.632	2.339	**UP**	Positive	
**IGF2R**	0.662	2.327	**UP**	Positive	
**SLC15A3**	0.687	2.313	**UP**	Positive	
**PIK3R5**	0.641	2.261	**UP**	Positive	
**ZBTB7B**	0.633	2.254	**UP**	Positive	
**ITPRIP**	0.642	2.211	**UP**	Positive	

Gene	ρ Spearman	log2FC	FC Management	Correlation with ANPEP	Functional Annotation
**AADAT**	-0.643	2.195	**UP**	Negative	
**NATD1**	0.641	2.181	**UP**	Positive	
**LRCH4**	0.631	2.169	**UP**	Positive	
**ARRB2**	0.666	2.158	**UP**	Positive	
**RNF166**	0.644	2.148	**UP**	Positive	
**ZNF746**	0.666	2.121	**UP**	Positive	
**FMNL1**	0.650	2.054	**UP**	Positive	
**RRP12**	0.636	2.029	**UP**	Positive	
**RPGRIP1L**	-0.653	-2.023	**DOWN**	Negative	
**MKNK2**	0.676	2.002	**UP**	Positive	
**PISD**	0.635	1.798	**UP**	Positive	
**PTPRE**	0.651	1.786	**UP**	Positive	
**LIMK2**	0.662	1.698	**UP**	Positive	
**PLEKHG3**	0.678	1.676	**UP**	Positive	
**RHOG**	0.652	1.623	**UP**	Positive	
**DUSP1**	0.645	1.589	**UP**	Positive	
**AMPD2**	0.671	1.353	**UP**	Positive	

Gene	ρ Spearman	log2FC	FC Management	Correlation with ANPEP	Functional Annotation
**GPR180**	-0.631	1.311	**UP**	Negative	
**GRK6**	0.658	1.309	**UP**	Positive	
**CSK**	0.667	1.218	**UP**	Positive	
**KLF13**	0.671	-1.177	**DOWN**	Positive	
**ITPK1**	0.635	1.165	**UP**	Positive	
**SLC43A2**	0.664	1.041	**UP**	Positive	
**PGS1**	0.684	1.007	**UP**	Positive	
